# Fine-scale detection of population-specific linkage disequilibrium using haplotype entropy in the human genome

**DOI:** 10.1186/1471-2156-11-27

**Published:** 2010-04-23

**Authors:** Hideaki Mizuno, Gurinder Atwal, Haijian Wang, Arnold J Levine, Alexei Vazquez

**Affiliations:** 1 The Simons Center for Systems Biology, Institute for Advanced Study, Princeton, New Jersey, USA; 2 Pharmaceutical Technology Department, Chugai Pharmaceutical Co. Ltd., Kamakura, Kanagawa, Japan; 3 Department of Biosciences and Bioinformatics, Kyushu Institute of Technology, Iizuka, Fukuoka, Japan; 4 Quantitative biology, Cold Spring Harbor Laboratory, Cold Spring Harbor, New York, USA; 5 Laboratory of Systems Biology, Fudan University, Shanghai, PR China; 6 The Cancer Institute of New Jersey, New Brunswick, New Jersey, USA; 7 Department of Radiation Oncology, UMDNJ-Robert Wood Johnson Medical School, New Brunswick, New Jersey, USA

## Abstract

**Background:**

The creation of a coherent genomic map of recent selection is one of the greatest challenges towards a better understanding of human evolution and the identification of functional genetic variants. Several methods have been proposed to detect linkage disequilibrium (LD), which is indicative of natural selection, from genome-wide profiles of common genetic variations but are designed for large regions.

**Results:**

To find population-specific LD within small regions, we have devised an entropy-based method that utilizes differences in haplotype frequency between populations. The method has the advantages of incorporating multilocus association, conciliation with low allele frequencies, and independence from allele polarity, which are ideal for short haplotype analysis. The comparison of HapMap SNPs data from African and Caucasian populations with a median resolution size of ~23 kb gave us novel candidates as well as known selection targets. Enrichment analysis for the yielded genes showed associations with diverse diseases such as cardiovascular, immunological, neurological, and skeletal and muscular diseases. A possible scenario for a selective force is discussed. In addition, we have developed a web interface (ENIGMA, available at http://gibk21.bse.kyutech.ac.jp/ENIGMA/index.html), which allows researchers to query their regions of interest for population-specific LD.

**Conclusion:**

The haplotype entropy method is powerful for detecting population-specific LD embedded in short regions and should contribute to further studies aiming to decipher the evolutionary histories of modern humans.

## Background

Modern humans emerged in Africa approximately 200,000 years ago and over the last 100,000 years dispersed around the world adapting to different environments [1]. The evolutionary histories during this period are reflected in the human genome by "selective sweeps" wherein beneficial alleles keep the genetic patterns of the surrounding sites [1,2]. The recent availability of high density maps of single nucleotide polymorphisms (SNPs) has provided us with a unique opportunity to uncover such selection traits.

Classically, statistical measurements such as *r*^2 ^and *D*' that test linkage disequilibrium (LD) at the resolution of two SNPs have been used to detect regions that have undergone recent selection [2]. However, their pairwise fashion cannot capture multilocus associations and so their testing power is limited [3]. Newly developed techniques, which are based on the concept of extended haplotype homozygosity (EHH) (e.g. LRH, iHS, XP-EHH) [4-6] and the composite likelihood ratio (CLR) [7], incorporate multilocus association and show higher power than conventional statistics. Nevertheless, those methods are weak in handling low SNP counts from minor alleles and/or require allele polarity (ancestral/derived), making their scores less reliable. Further, they need a relatively large window size to distinguish signal from noise, and so the human genome has not been investigated at resolution below 100 kb. Considering that recombination hotspots are estimated to exist with a frequency of at least one every 60 kb [8,9] and erode LD, genomic scans of short/intermediate resolution would give more detailed insight into recent human evolution [10].

Lately, entropy of haplotype frequency has been proposed as a general measure to quantify the strength of LD and thus to uncover evolutionary forces [3,11]. By definition, the haplotype entropy incorporates multilocus association, is proficient at handling low allele frequencies and does not rely on allele polarity. These features enable us to fully utilize nucleotide information and make short haplotype analysis feasible. In this study, we report a fine-scale genomic scan for population-specific LD, which is indicative of natural selection, using haplotype entropy.

## Results

### Haplotype entropy for detecting population-specific LD

Entropy is an established measure of diversity or information content. Here we use entropy to quantify the genetic diversity of given haplotypes as introduced by Nothnagel *et al*. [3] and Atwal *et al*. [11]. The analysis begins by counting the number of each haplotype within the genomic region of interest. Using this information on frequency of haplotype, we compute its entropy (see Methods). Low entropy is associated with low genetic diversity, where one or a few haplotypes are over-represented at high frequency in the region. On the other hand, high entropy is indicative of high genetic diversity, where various haplotypes are equally represented at small frequencies in the region. Under neutrality, stochastic processes such as mutation, recombination and genetic drift perturb genetic variation of the genome. Meanwhile, advantageous alleles keep the genetic pattern of linked sites by "selective sweep" [1,2] which decreases observations of recombination and increases the frequency of certain haplotypes, leading to low haplotype entropy. This suggests that the regions with entropy distinct from what is expected under neutral evolution are candidate targets for natural selection.

The original haplotype entropy method resorted to theoretical formula [3] or simulation [11] to estimate expected haplotype entropy of neutral evolution. However, these methods require a vast amount of calculations and reasonable parameters of the local recombination rate for each SNP, limiting the haplotype entropy method to a genome-wide application. Alternatively, we can compare two populations, where the entropy from one population provides the reference of the neutral evolution for the other. This comparison is also beneficial because it virtually cancels the effect of the physical distance between SNPs for which haplotype entropy does not take into account. In this approach, population-specific LDs are identified by extreme entropy differences in certain genomic regions between populations. This modification maintains the key features that are ideal for short haplotype analysis: the incorporation of multilocus association, conciliation with low allele frequencies, and independence from allele polarity.

### Simulation

**Figure 1:**
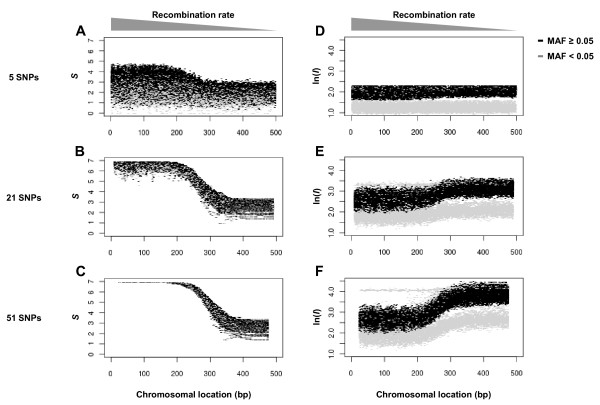
**Plots for *S *and *I *along model chromosomes**. (A-C) Model chromosomes having a gradient recombination rate were created and scanned for haplotype entropy (*S*) with window size of 5, 21 and 51 loci. x-axis represents locus position and y-axis represents magnitude of *S*. (D-F) The same chromosomes were scanned for integrated EHH (*I*). Because *I *is used in log-scale to give XP-EHH, log-transformed *I *was plotted. SNPs with MAF < 0.05 are indicated in gray.

First, we illustrate the ability of the method to present difference in LD strength in a simulation study. For this purpose, we prepared model chromosomes with a continuous gradient recombination rate from one end to the other end (See Methods). Then haplotype entropy (*S*) was scanned using three different window sizes: 5, 21 and 51 loci. As expected, *S *was elevated at high-rate recombination sites and was lower at low-rate recombination sites (Figure 1A-C). More importantly, the differences in entropy between high- and low-rate recombination sites were considerably larger than the variations in entropy at the site of the same recombination rate when a window size was 21 loci or larger. This result indicated that the method can distinguish differences in LD strength from random noise using short haplotypes such as 21 SNPs.

Next, for comparison, we scanned integrated EHH (*I*), a representative measurement for multilocus LD to give XP-EHH [6], for each SNP. *I*, which is designed to give high scores when LD is strong, was low at high-rate recombination sites and high at low-rate recombination sites (Figure 1D-F). As previous studies have noted, *I *presented highly skewed scores when a core SNP had a low minor allele frequency (MAF) (Figure 1D-F, gray dots) [5,6,12]. Note that, even when we ignored low MAF SNPs, *I *was still noisier compared to *S *and required a large window size (e.g. 51 SNPs) to secure reliable discrimination power. The observation level was robust, even when examined at a 100-fold higher mutation rate (Additional file 1). These results suggested the ability of *S *to better quantify the strength of LD with high resolution. We also note that, the feature of conciliation with low allele frequencies (Figure 1A-C, gray dots) would keep *S *robust against stochastic perturbagens such as recent mutations and yin-yang haplotypes [13] and artificial noise such as genotyping errors and haplotype inference errors.

### Analysis of the HapMap dataset

**Figure 2:**
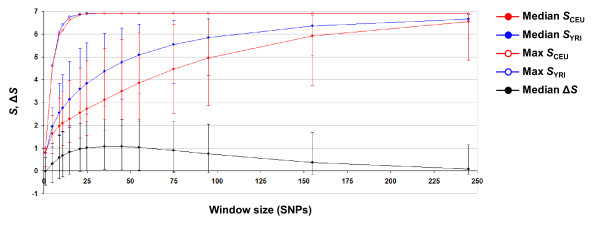
**Relationship between window size and entropy for HapMap data**. Filled circles represent medians of *S *distribution for CEU (red), YRI (blue) and the difference between them (Δ*S*, black) with 95% confidence intervals. Open circles represent maximum entropy for both CEU and YRI populations.

We analyzed 1,969,724 SNPs for two distinct populations from the HapMap project [14], the Yoruba in Ibadan, Nigeria (YRI) and residents of Utah with European ancestry (CEU). The first critical point at this stage was the choice of window size. Too small a window resulting in little haplotype diversity would reduce the power to distinguish differences in LD from random noise. On the other hand, too large a window differentiates all haplotypes in the region and causes entropy saturation. In addition, we wanted the window size to be considerably shorter than that of previous studies (100 kb) in order to target a fine-scale genomic scan. Thus, we investigated the HapMap dataset with respect to the relationship between window size and haplotype entropy as well as segment length. On average, haplotype entropy (*S*) saturation occurred at around 200 SNPs for both the YRI and CEU populations, with some regions achieving saturation at 25 SNPs (Figure 2). The difference in entropy between the two populations (Δ*S*) peaked at a window size between 20-50 SNPs. This led us to choose a window size comprised of 21 SNPs, satisfying the requirements of no entropy saturation and sufficient entropy difference. The corresponding segments had median lengths of 22.3 kb; about 99% were shorter than 100 kb (Additional file 2). At this window size, the segment length had limited influence on *S *(Additional file 3). In addition, we confirmed that *S *actually had a smoother pattern than *I *in this dataset (Additional file 4).

**Figure 3:**
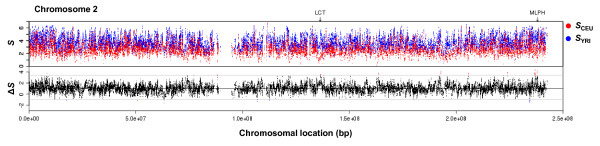
**Plots for *S*_YRI_, *S*_CEU _and Δ*S *along chromosomal location**. The entire chromosome 2 is shown. The upper panel shows entropy for both CEU (red) and YRI (blue) along the chromosomal location. The lower panel presents the entropy difference between two populations. The bold center line indicates the median of genome-wide distribution and the outer lines indicate the 0.1% tails. SNPs in the tails are shown for the CEU signature (red) and for the YRI signature (blue). Arrows indicate the *LCT *and *MLPH *gene regions.

We next focused on the differences between the CEU and YRI populations. The YRI exhibited greater haplotype entropy across the genome than the CEU (Figure 3, upper panel; Mann-Whitney U test *p*-value < 2.2e-16; see also Additional file 5), consistent with the concept that Africans are ancestral and have more genetic variation [1]. The differences in haplotype entropy between two populations were calculated for each SNP (Figure 3, lower panel). Because there are several uncertainties in our current knowledge for population genetics models [1,2], we estimated their statistical significance empirically. The empirical approach takes demographics into account and can be used even when accurate parameters are not available [15]. Based on the assumption that most of the genome is neutral for differentiating two populations, the extreme cases in the 0.1% tails of the genome-wide distribution (Additional file 5) were selected as candidate population-specific LD signatures. Mapping the selected SNPs to NCBI genes yielded 150 genes for CEU and 170 for YRI (Additional files 6 and 7). The signatures were enriched in genes detected in previous genome-wide studies [5,6,12,16] with high statistical significance (Table 1). On the other hand, there were also considerable non-overlapping fractions, suggesting different detection powers among the haplotype entropy method and other methods. For example, our signatures missed a well-established case of *LCT *[2], a lactose tolerance gene selected in the CEU population. Haplotype entropy around this gene for CEU was very low across the long region (Additional file 8). However, that of YRI was also moderately low. As a result, the entropy differences over the short range were not large enough to be captured, in contrast to strong iHS signals for CEU. To detect long-range LD such as the *LCT *region, other methods, for example EHH derivatives [5,6], would be more suitable.

**Table 1:** Comparison with previously reported signatures

					Overlap to haplotype entropy method			
						
Method	Scan resolution	Dataset	Population	Gene #	Expected	Observed	***χ***^2^***p*-value**	Ref.
Entropy	23 kb	HapMap2	Caucasian	150	-	-	-	
CLR	~500 kb	Perlegen	Caucasian	41	0.2896	5	3.8e-15	[16]
iHS	100 kb	HapMap2	Caucasian	246	1.7374	13	< 2.2e-16	[5]
iHS	200 kb	HGPD	Caucasian	194	1.3701	12	< 2.2e-16	[12]
XP-EHH	~800 kb-3.5 Mb	HapMap2	Caucasian	23	0.1624	8	< 2.2e-16	[6]
XP-EHH	~500 kb	HGPD	Caucasian	177	1.2501	22	< 2.2e-16	[12]
								
Entropy	23 kb	HapMap2	African	170	-	-	-	
CLR	~500 kb	Perlegen	African	10	0.0800	2	4.6e-07	[16]
iHS	200 kb	HGPD	African (Bantu)	245	1.9610	14	< 2.2e-16	[12]
iHS	200 kb	HGPD	African (Biaka)	165	1.3207	4	0.05595	[12]
iHS	100 kb	HapMap2	African	262	2.0971	15	< 2.2e-16	[5]
XP-EHH	200 kb	HGPD	African (Bantu)	400	3.2017	27	< 2.2e-16	[12]
XP-EHH	200 kb	HGPD	African (Biaka)	425	3.4018	7	0.08843	[12]
XP-EHH	~800 kb-3.5 Mb	HapMap2	African	ND	-	-	-	[6]

### Characteristics of population-specific LD signatures

**Figure 4:**
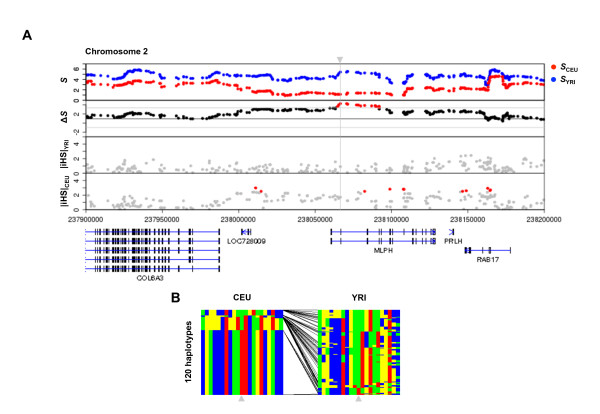
**Plots for the *MLPH *gene region and haplotype structures around the *MLPH rs12465081 *locus**. (A) The first and second panels show the haplotype entropy and entropy difference, respectively, the same as in Figure 3. The third and fourth panels show the iHS scores for YRI and CEU populations. SNPs with |iHS| > 2.5 (corresponding to the top 1%) are shown for CEU (red) and YRI (blue). Gene structures of the region are indicated at the bottom. The gray arrowhead indicates the *rs12465081 *locus. (B) Haplotype structures around the *MLPH rs12465081 *locus (gray arrowhead) for two populations are shown. In each population panel, rows represent haplotypes and columns represent loci with color-coded nucleotides, A: red, T: blue, G: green, and C: yellow. Lines connecting the two panels indicate changes in frequency of each haplotype in the two populations.

**Figure 5:**
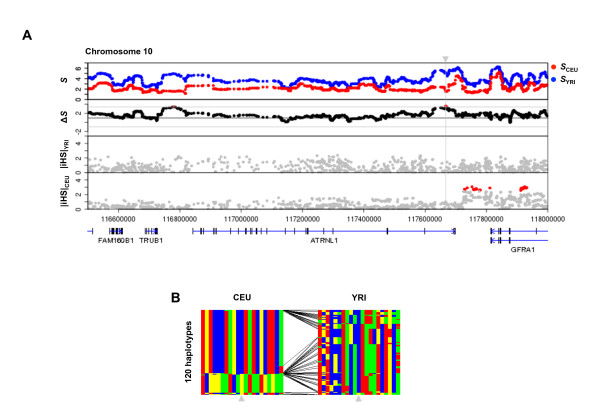
**Plots for the *ATRNL1 *gene region and haplotype structures surrounding *rs17354968 *locus**. (A) and (B) are the same as Figure 4.

We looked into the characteristics of population-specific LD signatures, which are potential targets of natural selection, from the viewpoint of pigmentation because it is one of the most conspicuous features differentiating two populations, and conventional genome-wide studies have analyzed the associated genes [5,12,16]. In Parra's review, 10 pigmentation genes were listed as candidate targets of natural selection [17]. Among them, three genes (*OCA2*, *SLC24A5 *and *SLC45A2*) were of particular interest because of their association with normal pigmentation variation [17]. Two of the three, *OCA2 *and *SLC24A5*, were included in the CEU signature with high significance (Additional file 6, empirical *p *= 0.00077 and 0.00042, respectively). Although not in the 0.1% threshold, *SLC45A2 *also scored high (*p *= 0.00412). Regarding the other constituents of the list, our scan detected the signal for *KITLG *in its 35 kb downstream for CEU, in contrast to iHS signals in the coding region (Additional file 9). This observation is consistent with a previous study using CLR [16], indicating a signal peak downstream of *KITLG *for Caucasians rather than in the coding region. In addition, haplotype structure suggested that short-range genetic diversity is greater downstream of *KITLG *than in the coding region (Additional file 9). *ADAM17 *and *ADAMTS20*, candidate genes for Asian populations, were not detected, as expected, and the other 4 genes did not show particular signals. Besides the genes in Parra's list, we noticed that *MLPH*, a component of the melanosome transport machinery [18,19], was included in the CEU signature with one of the most extreme *p*-values (Figure 4, *p *= 6.62e-6). This gene had not been reported in any genome-wide studies until recently when Pickrell *et al*., using the newly-released Human Genome Diversity-CEPH Panel (HGPD) dataset, detected it as a new candidate for recent selection in non-African populations [12]. The fact that we extracted a result consistent with Pickrell *et al*. from the HapMap dataset indicates that the haplotype entropy method has unique detection abilities over other methods that have been applied. Further, we found another new candidate, *ATRNL1*, a homolog of pigmentation-related gene *ATRN*, in the CEU signature (Figure 5A, *p *= 0.00044). *ATRNL1 *has been shown recently to compensate for pigmentation alteration in the *ATRN *null mouse [20]. Although *ATRN *did not show a distinct signal, a clear contrast of haplotype diversity between CEU and YRI indicated existence of an evolutionary force on *ATRNL1 *(Figure 5B). Our new finding of *ATRNL1 *as a novel candidate of population-specific LD may promote further study into its evolutionary involvement in human pigmentation, as was found with *SLC24A5 *[2,17].

To investigate insights from signatures other than pigmentation, we used Ingenuity Pathway Analysis (IPA) [21], which enables assessment of the enrichment of genes in specific functional categories and diseases. In both CEU and YRI signatures, diverse disease categories such as cardiovascular, immunological, neurological, and skeletal and muscular diseases showed high associations (Table 2). This result attracted us because a number of individual studies have discovered population-specific loci susceptibility to, for example, cardiovascular diseases [10,22], schizophrenia [23], Crohn's disease [24-26] and diabetes [12,26,27], and have suggested they are consequences of natural selection. In addition, although the underlying mechanism has not been clarified, some previous genome-wide studies have reported an association between recent selection and the biological functions of skeletal development, brain development, and immune response [5,16].

**Table 2:** Functions and diseases related to both CEU and YRI signatures

	CEU	YRI
**Diabetes**	1.70E-17	2.70E-20
**Coronary artery disease**	3.78E-17	3.12E-13
**Genetic disorder**	1.09E-14	4.53E-14
**Cardiovascular disease**	1.34E-14	4.78E-11
**Non-insulin-dependent diabetes mellitus**	1.03E-12	8.66E-15
**Endocrine system disorder**	1.91E-12	1.25E-18
**Hypertension**	2.13E-12	2.83E-11
**Neurological disorder**	2.49E-12	3.27E-12
**Crohn's disease**	7.59E-12	7.25E-09
**Bipolar affective disease**	3.50E-11	5.24E-09
**Metabolic disorder**	5.66E-11	9.83E-16
**Inflammatory disorder**	2.56E-09	1.08E-08
**Digestive system disorder**	2.66E-09	1.31E-07
**Skeletal and muscular disorder**	1.49E-08	1.58E-08
**Progressive motor neuropathy**	2.53E-08	5.59E-12
**Autoimmune disease**	7.28E-08	1.09E-12
**Immunological disorder**	2.46E-07	3.66E-13
**Rheumatoid arthritis**	3.78E-07	2.54E-07
**Shape change of epithelial cells**	8.86E-07	
**Alzheimer's disease**	3.60E-06	2.27E-07
**Parkinson's disease**	7.24E-06	5.29E-06
**Shape change of dermal cells**	9.33E-06	
**Insulin-dependent diabetes mellitus**		3.76E-15
**Neuropathy**		5.38E-12
**Amyotrophic lateral sclerosis**		1.36E-09
**Arthritis**		1.44E-07

### Vitamin D hypothesis

An interesting hypothesis has been proposed by McGrace [28] claiming that low prenatal vitamin D increases the risk of a wide range of diseases such as multiple sclerosis, diabetes, schizophrenia, prostate cancer, breast cancer and colorectal cancer because of its versatile function in normal development. Additional circumstantial evidence encouraged us to integrate this "vitamin D hypothesis" and population-specific selection. First, the assumed prime function of pigmentation, one of the most convincing as a recent selection target differentiating CEU and YRI populations, is to control vitamin D synthesis from ultra violet exposure [17,29]. Second, in addition to McGrace's list of diseases, animal model studies and epidemiological surveys have further linked vitamin D insufficiency to cardiovascular disease and inflammatory bowel disease [17,30,31], as well as abnormal brain development [31]. These diseases were dominant in our enrichment analysis, with the exception of cancer, which may have less impact on natural selection due to its relatively late onset in life [17,32,33].

To assess this possibility, we looked into the entropy of the *VDR *gene, a vitamin D receptor. Although it did not reach the 0.1% criteria, the difference in entropy of the 3' region of *VDR *between CEU and YRI was relatively high (Additional file 10, *p *= 0.00916). The corresponding haplotype contained the loci *BsmI*, *Tru9I*, *Ap*a*I *and *TaqI *(Additional file 10), whose polymorphisms have been shown to associate with hypertension, coronary artery disease, Crohn's disease, diabetes, multiple sclerosis, Alzheimer's disease, cognition, and depression [31,34-36]. We also found that *RXRA*, a heterodimeric partner of *VDR*, also showed borderline significance (Additional file 11, *p *= 0.00139). These results are suggestive that vitamin D might have been involved in the alteration of risk of disease and abnormal development and consequently in the genetic adaptation of modern humans, but validation from further study is necessary.

## Discussion and conclusion

In this study, we presented a fine-scale genomic scan using haplotype entropy to detect population-specific LD, which is indicative of natural selection, in the human genome. The yielded signatures included a number of previously detected genes, and overlaps with previously reported signatures were significant. On the other hand, there were a considerable number of novel predictions. These inconsistencies among the methods can be attributed to several factors.

First, the haplotype entropy method is effective to detect signals embedded in short regions with a window size such as 23 kb (21 SNPs), whereas previous methods had to contend with a window size of 100 kb or larger [5,6,12,16] to yield reliable signals. This high resolution allowed us to detect novel candidate regions such as *MLPH *and *ATRNL1 *that were undetected using previous methods. However, the haplotype entropy method has the issue of entropy saturation. Thus, under the constraint of a fixed window size, it cannot detect regions which may require a larger window size to show a sufficient signal such as *LCT*.

The second factor affecting detection is the use of a reference population. The practical limitations regarding computational resources and uncertainty in parameters drove us to compare two populations, where one population provides the reference of neutral evolution for the other and so regions showing highly different haplotype entropy between the populations can be deemed to be population-specific LD. Although this would allow us to detect "fixed" selection signatures in one population which cannot be found with intrapopulation methods [4,5], it still would cause false negatives when the regions have been selected in parallel in both populations [6]. This problem can be tackled by considering more than one population, which would provide a better neutral control and improve the ability of the method to uncover unusual LD regions in a specific population.

A third factor leading to non-overlapping signatures could be statistical error. Teshima *et al*. has shown that the empirical approach is reasonable but can cause a large number of false negatives [15]. Although we have focused on the genomic regions showing extreme entropy difference (top 0.1%), we cannot exclude the possibility that other signatures remain below the threshold. Since EHH derivatives and CLR are also based on the empirical approach, some inconsistencies may have been due to this limitation. Also, false positives need to be considered. Our analysis of simulation and HapMap datasets showed *I *was more variable than *S *(Figure 1 and Additional files 1 and 4). Thus, it is possible that earlier methods detected some false positives that the entropy method does not. At the same time, haplotype entropy method may have caused some false positives absent in previous signatures because it relied on much less information due to the smaller window size.

Therefore, although powerful, the haplotype entropy method is not an ultimate solution. Rather, it would be most effective as a complement to other methods. Its unique detection power can fill the gap between pairwise methods and new technologies such as EHH and CLR. It should also help in cross-validating candidates of natural selection from those statistics. We provide a web interface (ENIGMA at http://gibk21.bse.kyutech.ac.jp/ENIGMA/index.html) so that researchers can query their regions of interest in our fine-scale map of Caucasian and African population-specific LDs. Our works, taken together, would contribute to further studies towards understanding human evolution inscribed in the human genome.

## Methods

### Datasets

The HapMap2 release #24, a dataset of phased 1,969,416 SNPs, was downloaded from the project web site [14,37]. In this study, two populations each consisting of 120 chromosomes from 60 donors, were analyzed: Yoruba in Ibadan, Nigeria (YRI) and a group of residents of Utah with European ancestry (CEU). A data table from NCBI Build 36 was also obtained from the NCBI FTP site [38] and transcribed regions were considered for mapping the SNPs to genes.

### Genomic scan using haplotype entropy

The degree of genetic diversity was measured using the entropy (*S*) for haplotype frequency, *S *= - ∑ *p*(*i*)log_2_*p*(*i*), where *i *is an index of the haplotypes and *p*(*i*) is the frequency of haplotype *i *in the population. *S *achieves maximum score log_2_(*n*) when the given *n *haplotypes for the region differ from each other. *S *is 0 when all haplotypes are identical. Entropy difference Δ*S *was defined as Δ*S *= *S*_pop1 _- *S*_pop2_, where *S*_pop1 _and *S*_pop2 _are the haplotype entropies for two populations. For the genomic scan on the HapMap dataset, a window size of 21 SNPs was chosen for haplotype composition because it fulfilled three requirements: no entropy saturation, sufficient entropy difference and high resolution (See Results). SNPs on sex chromosomes and haplotypes for long segments (> 200 kb) were excluded. For each SNP, the Δ*S *between CEU and YRI was calculated and its empirical significance in the genome-wide distribution was determined. No correction operation for multiple testing was applied. A genomic scan for integrated EHH (*I*) was also done for chromosome 1 using the same window size of 21 SNPs. For the direct comparison to *S*, segment lengths of the haplotypes were not considered. iHS scores, the other EHH-based measurement, were downloaded from the Haplotter database [5,39].

### Simulation

We considered model chromosomes composed of 500 loci, where the *j*^th ^(1 ≤ *j *≤ 500) locus has recombination rate *r *= exp(- *j*/25) per haploid and generation, shaping a continuous gradient from one end (*r *= 1) to the other end (*r *= 2.1E-9). For each locus, initial allele frequency *k *and 1 - *k *(0 ≤ *k *≤ 1) for two alleles were randomly given. Using the GenomePop software [40], the evolutionary process was simulated for 5,000 generations with the parameter of population size as 10,000 and mutation rate per locus as 2.0E-9. Then, 120 chromosomes for 60 individuals were sampled from one run of the simulation and scanned for *S *and *I *using window sizes of 5, 21 and 51 loci. The simulation was repeated 100 times.

### Enrichment analysis

CEU and YRI signatures were queried against IPA version 7.5 [21]. "Function and Disease" libraries were overlaid on each signature and enrichment scores were calculated. Categories with *p *< 1E-5 significance were listed.

## Authors' contributions

HM and GA conceived and designed the statistical methods. HM, AJL and AV designed the project. HM and HW analyzed the data. HM, GA and AV participated in writing the manuscript. All authors read and approved the final manuscript.

## Supplementary Material

Additional file 1 **Plots for *S *and *I *for model chromosomes with a high mutation rate**. The model chromosomes were created to have a high mutation rate (2.0E-7 per locus per generation) and scanned for *S *and *I *in the same manner as Figure 1. Click here for file

Additional file 2 **Relationship between window size and segment length for HapMap data**. Segment lengths of the median (solid line) and the 99^th ^percentile (dotted line) for the different window sizes are plotted. Click here for file

Additional file 3 **Relationship between segment length and *S *at a window size of 21 SNPs for HapMap data**. Scatter plots of segment length and *S *at a window size of 21 SNPs were created. Upper panel is for YRI population, and lower panel is for CEU population. The Median (black line) and the 99^th ^percentile (gray line) of the segment lengths are indicated. Click here for file

Additional file 4 ***S *and *I *for HapMap data**. The first part of chromosome 1 for both CEU and YRI populations was scanned for *S *and *I *(window size = 21 SNPs). For ease of comparison with the general patterns for *S *and *I*, log-scale *I *is displayed upside down. SNPs with MAF < 0.05 are indicated in red. Click here for file

Additional file 5 **Histograms for *S*_YRI_, *S*_CEU _and Δ*S***. (A) Genome-wide distribution of *S *for the CEU (red) and YRI (blue) populations. (B) Genome-wide distribution of entropy differences between two populations. The median (black line) and 0.1% thresholds (gray lines) for both tails are indicated. Click here for file

Additional file 6 The 150 genes comprising the CEU-specific LD signature. Click here for file

Additional file 7 The 170 genes comprising the YRI-specific LD signature. Click here for file

Additional file 8 Plots for the *LCT *gene region and haplotype structures around the *LCT rs3739022 *locus. Click here for file

Additional file 9 **Plots for the *KITLG *gene region and haplotype structures around the *rs7312974 *and *rs1162374 *loci**. The *rs1162374 *locus (arrowhead) and the the *rs7312974 *locus (asterisk) are indicated. Click here for file

Additional file 10 **Plots for the *VDR *gene region and haplotype structures around the *rs7963776 *locus**. Click here for file

Additional file 11 Plots for the *RXRA *gene region and haplotype structures around the *RXRA rs4917353 *locus. Click here for file
