# Supplementary figures and images for: Fine-scale detection of population-specific linkage disequilibrium using haplotype entropy in the human genome
Source: BMC Genet. 2010 Apr 23;11:27. doi: 10.1186/1471-2156-11-27 (PMC2873552; doi:10.1186/1471-2156-11-27)

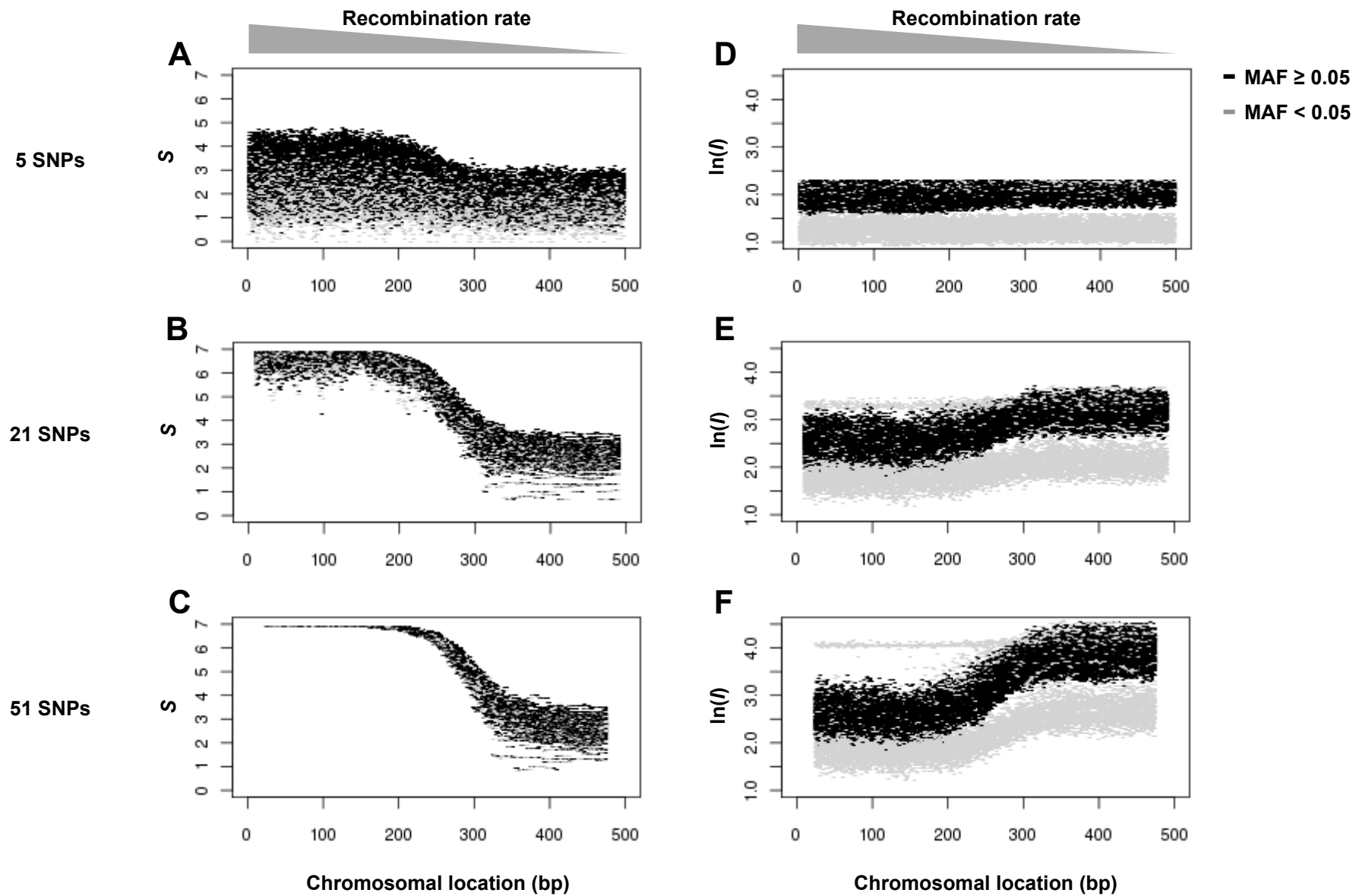

Supplement: Additional file 1 — Plots for S and I for model chromosomes with a high mutation rate. The model chromosomes were created to have a high mutation rate (2.0E-7 per locus per generation) and scanned for S and I in the same manner as Figure 1. [file 1471-2156-11-27-S1.PDF]

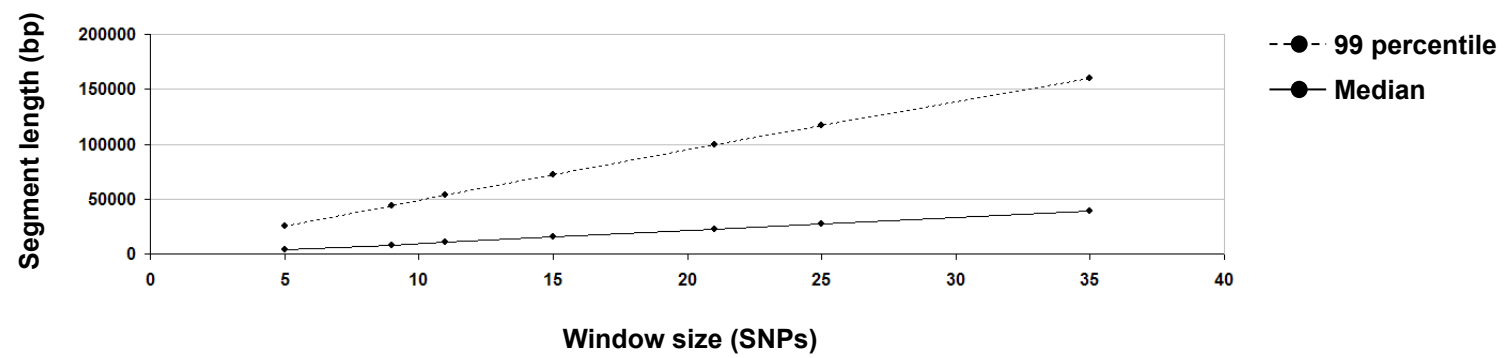

Supplement: Additional file 2 — Relationship between window size and segment length for HapMap data. Segment lengths of the median (solid line) and the 99th percentile (dotted line) for the different window sizes are plotted. [file 1471-2156-11-27-S2.PDF]

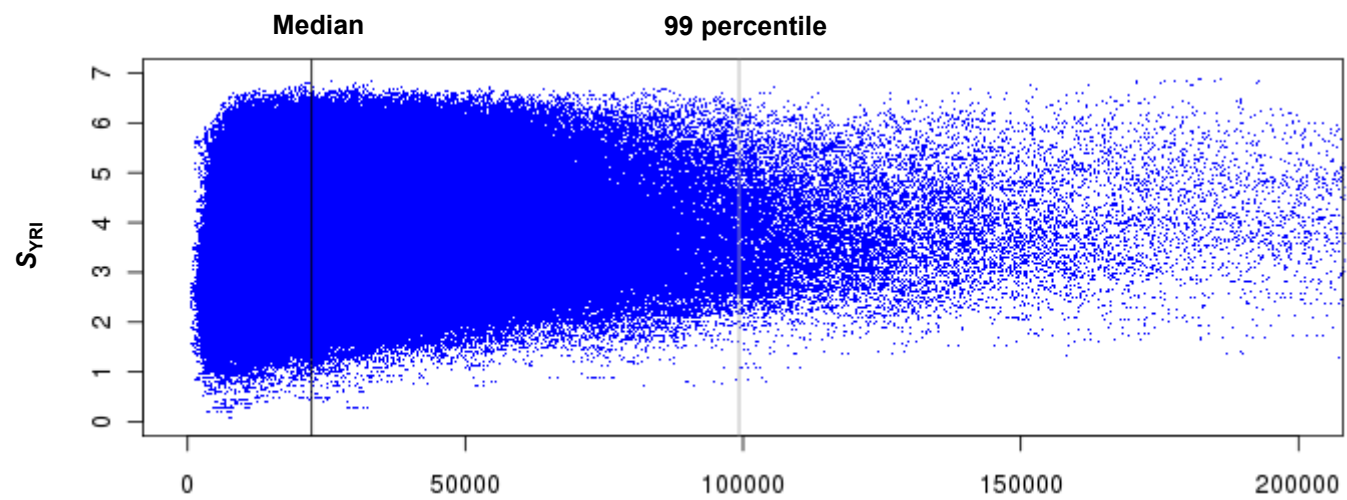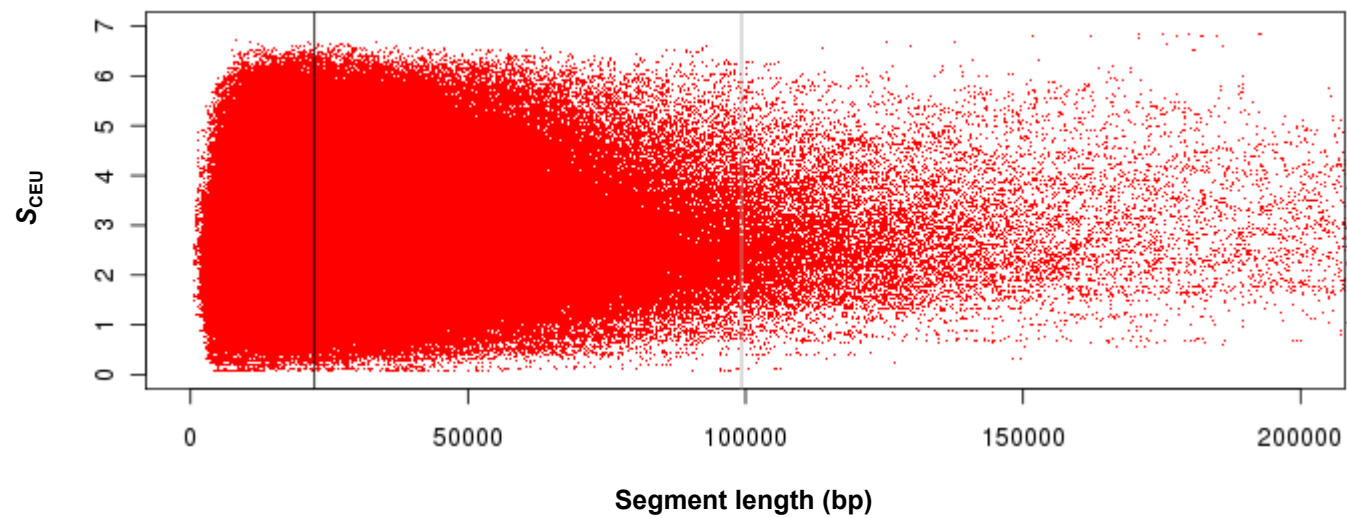

Supplement: Additional file 3 — Relationship between segment length and S at a window size of 21 SNPs for HapMap data. Scatter plots of segment length and S at a window size of 21 SNPs were created. Upper panel is for YRI population, and lower panel is for CEU population. The Median (black line) and the 99th percentile (gray line) of the segment lengths are indicated. [file 1471-2156-11-27-S3.PDF]

Chromosome 1

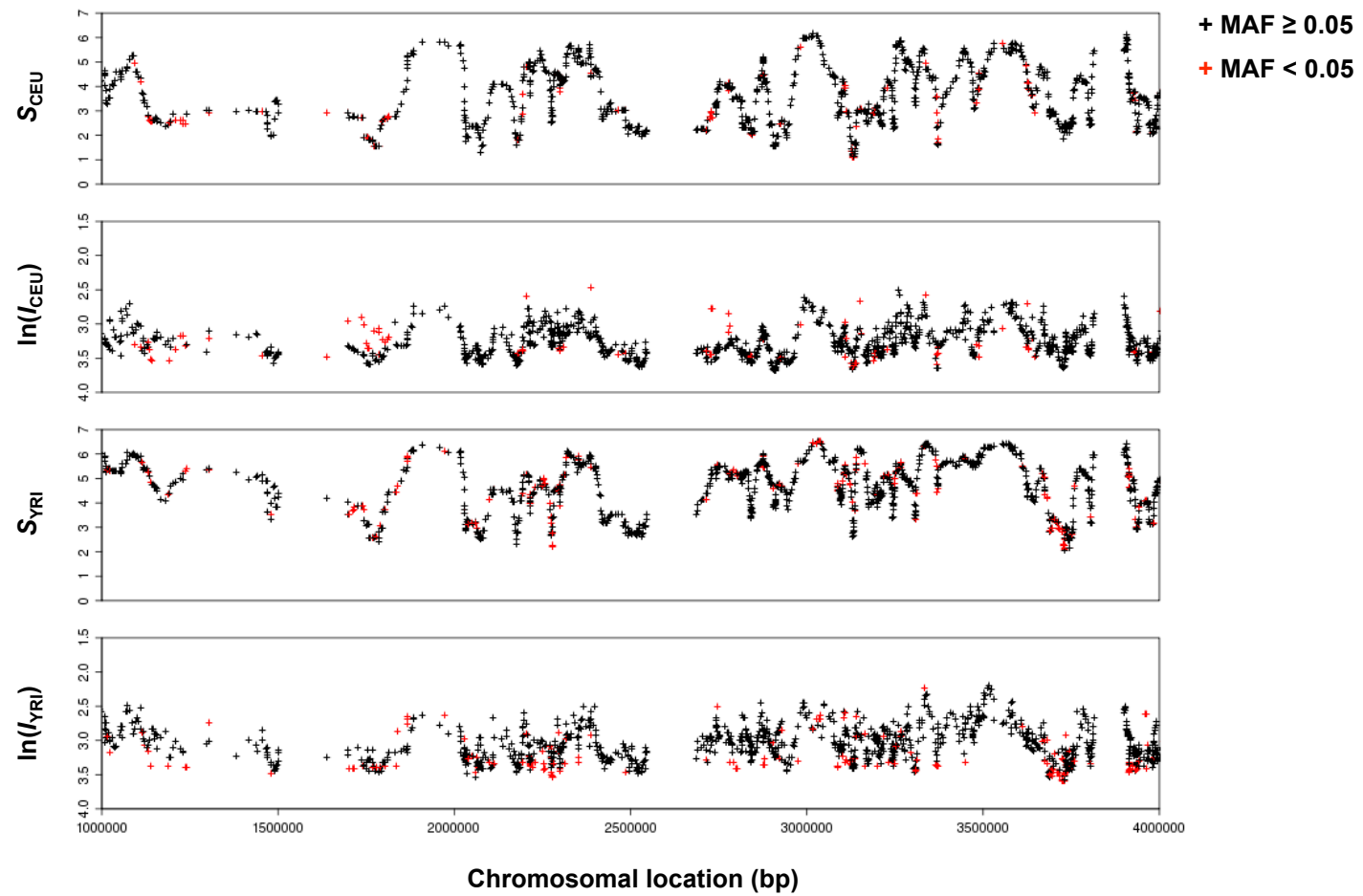

Supplement: Additional file 4 — S and I for HapMap data. The first part of chromosome 1 for both CEU and YRI populations was scanned for S and I (window size = 21 SNPs). For ease of comparison with the general patterns for S and I, log-scale I is displayed upside down. SNPs with MAF < 0.05 are indicated in red. [file 1471-2156-11-27-S4.PDF]

**A**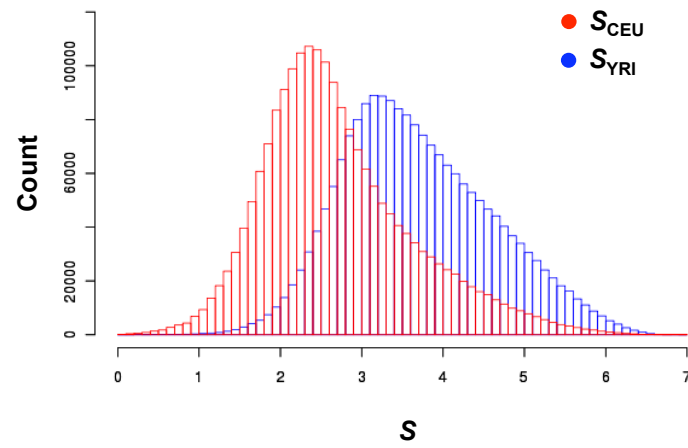**B**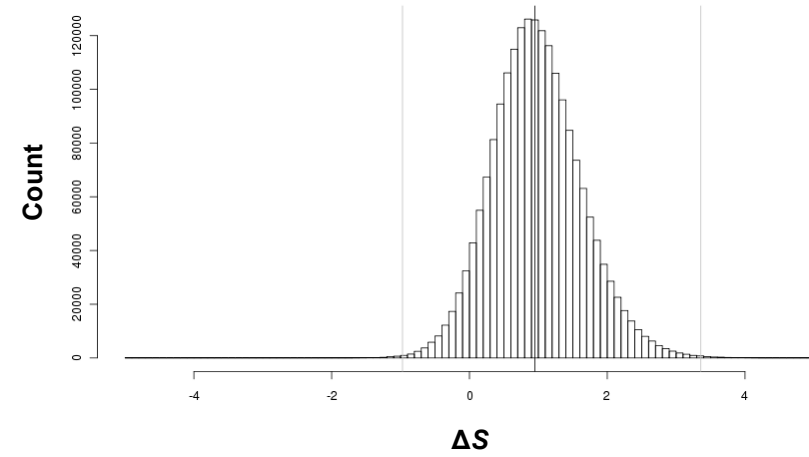

**Median = 0.9479453**

**99.9 percentile = 3.3588120**

**0.1 percentile = -0.9732882**

Supplement: Additional file 5 — Histograms for SYRI, SCEU and ΔS. (A) Genome-wide distribution of S for the CEU (red) and YRI (blue) populations. (B) Genome-wide distribution of entropy differences between two populations. The median (black line) and 0.1% thresholds (gray lines) for both tails are indicated. [file 1471-2156-11-27-S5.PDF]

**A**

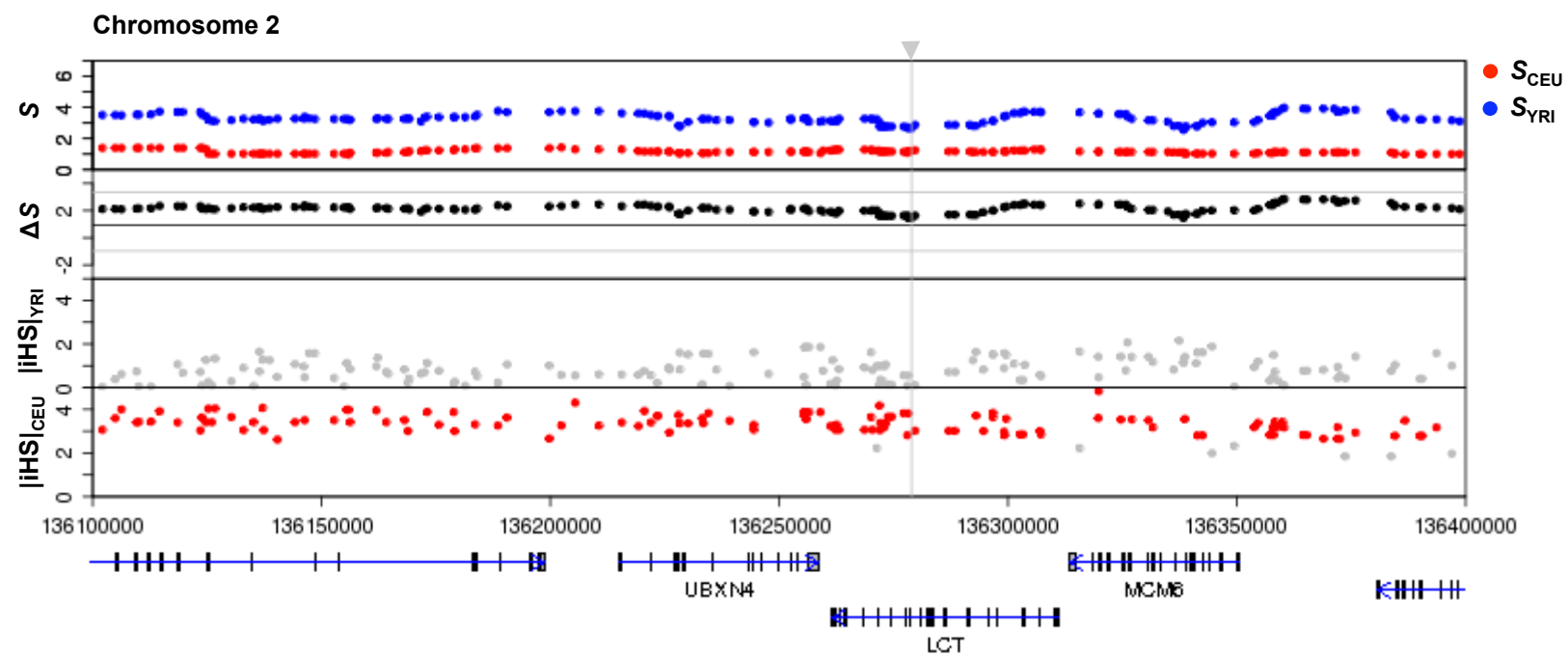

**B**

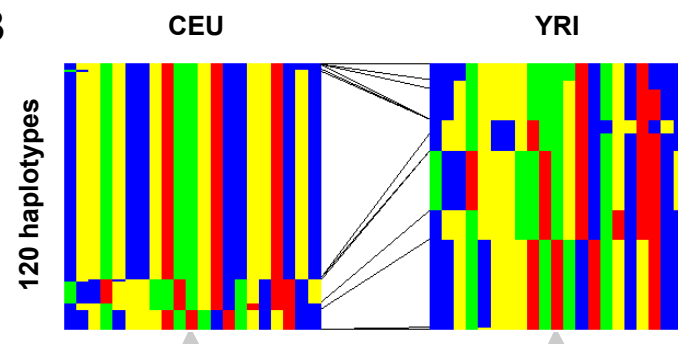

Supplement: Additional file 8 — Plots for the LCT gene region and haplotype structures around the LCT rs3739022 locus. [file 1471-2156-11-27-S8.PDF]

**A**

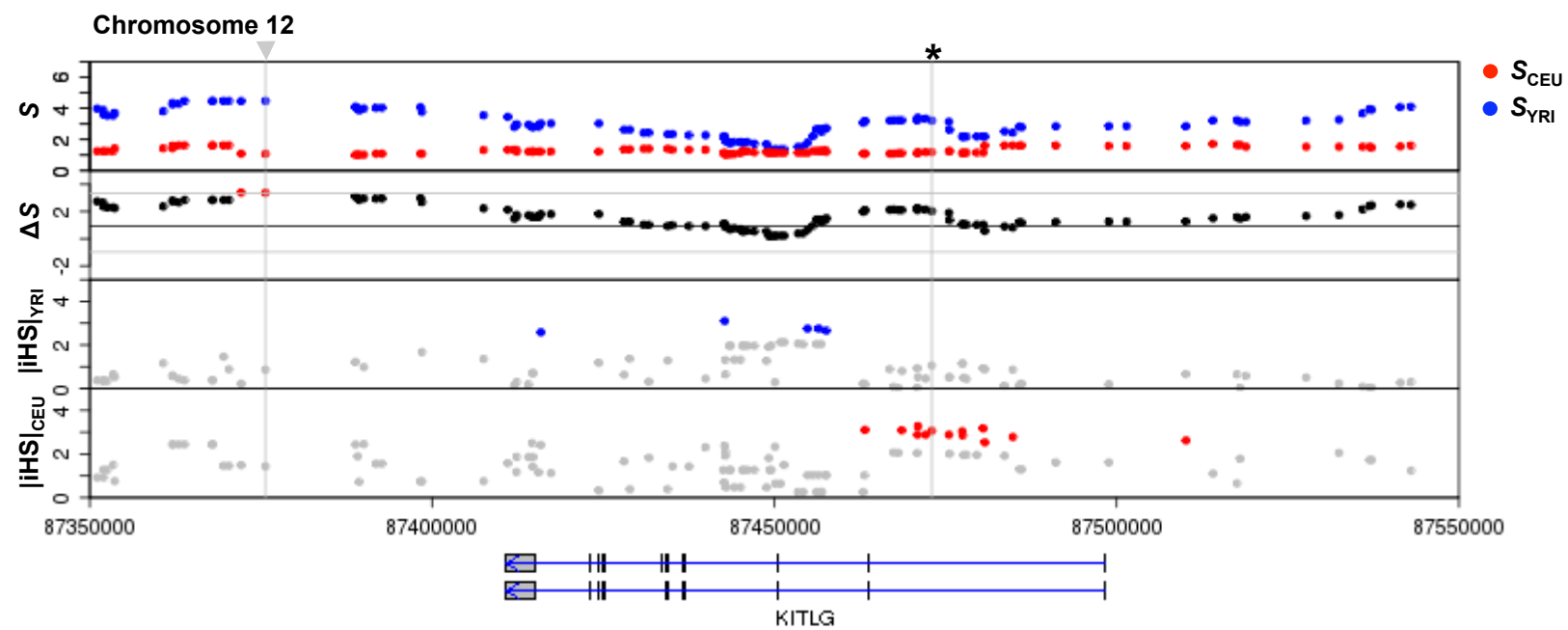

**B**

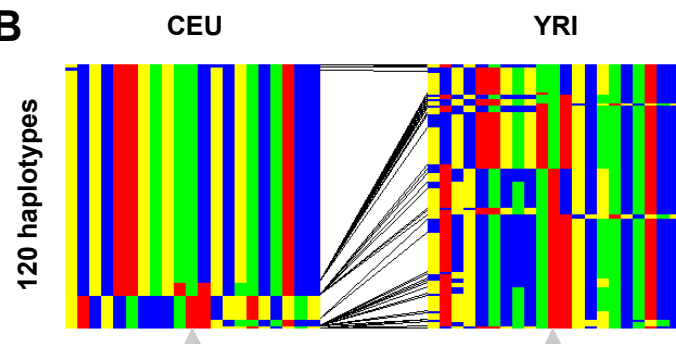

**C**

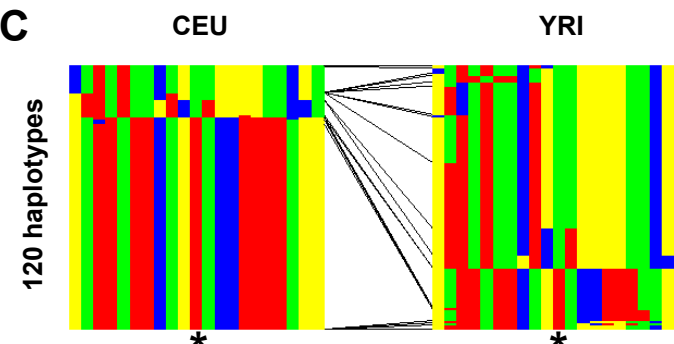

Supplement: Additional file 9 — Plots for the KITLG gene region and haplotype structures around the rs7312974 and rs1162374 loci. The rs1162374 locus (arrowhead) and the the rs7312974 locus (asterisk) are indicated. [file 1471-2156-11-27-S9.PDF]

**A**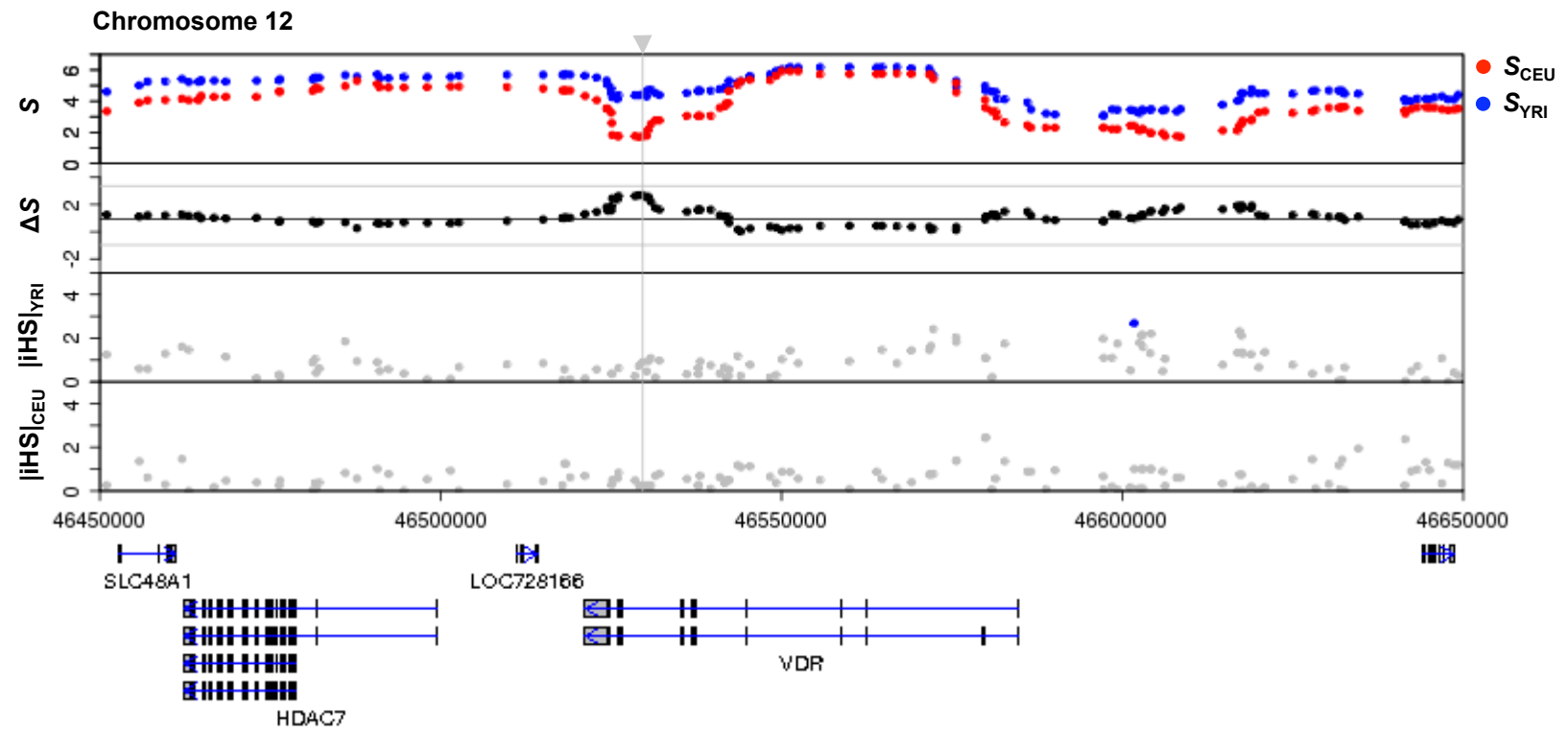**B**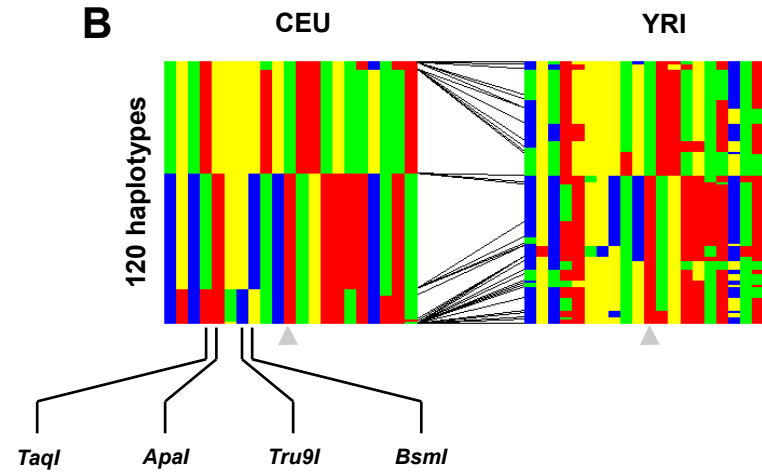

Supplement: Additional file 10 — Plots for the VDR gene region and haplotype structures around the rs7963776 locus. [file 1471-2156-11-27-S10.PDF]

**A**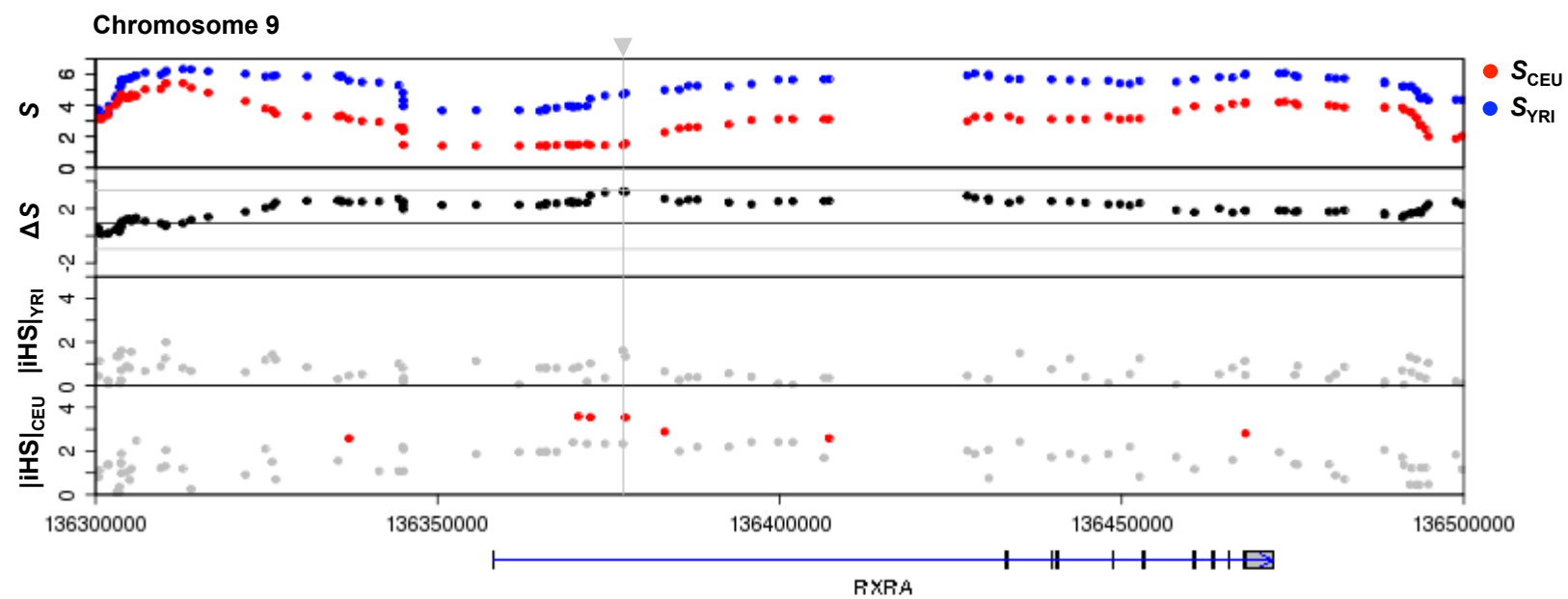**B**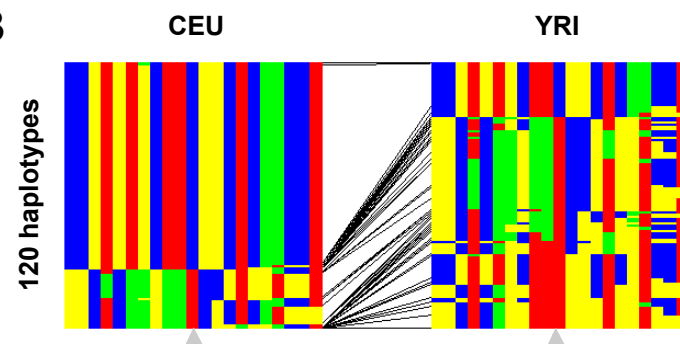

Supplement: Additional file 11 — Plots for the RXRA gene region and haplotype structures around the RXRA rs4917353 locus. [file 1471-2156-11-27-S11.PDF]
